# Региональная целевая программа «Профилактика йододефицитных заболеваний на 202Х–202Х годы» (Проект)

**DOI:** 10.14341/probl13120

**Published:** 2022-04-27

**Authors:** Е. А. Трошина, И. И. Дедов, Н. М. Платонова, Н. П. Маколина, И. М. Беловалова, Е. С. Сенюшкина, Г. А. Мельниченк

**Affiliations:** Национальный медицинский исследовательский центр эндокринологии; Национальный медицинский исследовательский центр эндокринологии; Национальный медицинский исследовательский центр эндокринологии; Национальный медицинский исследовательский центр эндокринологии; Национальный медицинский исследовательский центр эндокринологии; Национальный медицинский исследовательский центр эндокринологии; Национальный медицинский исследовательский центр эндокринологии

## Abstract

.

## ПРОЕКТ

## РЕГИОНАЛЬНАЯ ЦЕЛЕВАЯ ПРОГРАММА«ПРОФИЛАКТИКА ЙОДОДЕФИЦИТНЫХ ЗАБОЛЕВАНИЙ НА 202Х–202Х ГОДЫ»Субъект Российской Федерации (наименование)

## ПАСПОРТ ЦЕЛЕВОЙ ПРОГРАММЫ

Наименование Программы региональная целевая программа «Профилактика йододефицитных заболеваний на 202X–202X годы» Основание для решения о разработке Программы Постановление Правительства РФ от 26 декабря 2017 г. №1640 «Об утверждении государственной программы Российской Федерации “Развитие здравоохранения”» Постановление Правительства Российской Федерации от 05.10.1999 г. №1119 «О мерах по профилактике заболеваний, связанных с дефицитом йода» с изменениями и дополнениями от 11 июля 2020 г., 17 апреля 2021 г. Приказ Минздрава России от 15.01.2020 №8 «Об утверждении Стратегии формирования здорового образа жизни населения, профилактики и контроля неинфекционных заболеваний на период до 2025 года» Постановление Главного государственного санитарного врача РФ от 23.07.2008 №45 (ред. от 25.03.2019) «Об утверждении СанПиН 2.4.5.2409-08» (вместе с «СанПиН 2.4.5.2409-08. Санитарно-эпидемиологические требования к организации питания обучающихся в общеобразовательных учреждениях, учреждениях начального и среднего профессионального образования. Санитарно-эпидемиологические правила и нормативы»). Зарегистрировано в Минюсте России 07.08.2008 №12085 (с изм. и доп., вступ. в силу с 01.01.2020) Государственный заказчик Программы Высший исполнительный орган государственной власти субъекта Российской Федерации (наименование) или Орган исполнительной власти субъекта Российской Федерации в сфере здравоохранения (наименование) Основные разработчики Программы Специалисты Органа исполнительной власти субъекта Российской Федерации в сфере здравоохранения (наименование) Цель Программы Разработка и обеспечение функционирования системы профилактики, диагностики и лечения заболеваний, обусловленных дефицитом йода, на территории Российской Федерации Задачи Программы 1.Создание специализированной централизованной службы профилактики, диагностики и лечения йододефицитных заболеваний (ЙДЗ) в РФ в виде системы противозобных подразделений (кабинетов, центров), действующих на функциональной основе на базе лечебно-диагностических подразделений «головного» ЛПУ по профилю «эндокринология» субъекта РФ или регионального эндокринологического центра (диспансера, регионального эндокринологического центра). 2.Разработка и внедрение программы мероприятий по профилактике ЙДЗ в каждом муниципалитете субъекта РФ. 3.Усовершенствование статистического наблюдения за заболеваемостью населения болезнями, связанными с йодной недостаточностью, на уровне субъекта РФ. 4.Оснащение кабинетов (центров) профилактики и лечения ЙДЗ современным оборудованием, расходными материалами и лекарственными средствами. 5.Организация подготовки и повышения квалификации медицинского персонала по профилактике и лечению ЙДЗ. 6.Проведение на постоянной основе профилактических мероприятий, направленных на устранение ЙДЗ, и ведение мониторинга их эффективности в соответствии с разработанными стандартами. 7.Проведение научно-исследовательских работ, направленных на создание и внедрение новых методов профилактики, диагностики и лечения ЙДЗ. 8.Информационное обеспечение населения, медицинских работников соответствующими просветительскими и образовательными материалами по вопросам профилактики, диагностики и лечения заболеваний, обусловленных дефицитом йода. 9.Способствование насыщению рынка продовольственных товаров субъекта Российской Федерации (наименование) йодированной солью. Целевые индикаторы и показатели Программы Целевые индикаторы Программы 1.Охват территории РФ массовой профилактикой ЙДЗ. a.Количество центров (кабинетов) профилактики и лечения ЙДЗ (не менее 2 в каждом субъекте РФ). b.Количество функционирующих региональных сегментов реестра ЙДЗ (1 в каждом субъекте РФ). c.Охват территорий (доля, %) регулярным мониторингом эпидемиологической ситуации и эффективности лечебно-профилактических мероприятий. 2.Уровень оснащенности Центров (кабинетов) по профилактике и лечению ЙДЗ необходимым оборудованием (включая лабораторную технику и расходные материалы) и лекарственными средствами. Основные показатели эффективности профилактической программы в субъекте РФ. 3.Медиана экскреции йода с мочой (мкг/л). 4.Количество домохозяйств, употребляющих йодированную соль (доля, %). 5.Доля (%) неонатальной гипертиреотропинемии (уровень неонатального тиреотропного гормона (ТТГ) выше 5 мкМЕ/л). Дополнительные показатели эффективности профилактической программы в субъекте РФ. 6.Наличие зоба у детей возраста 8–10 лет (доля, %). 7.Показатель заболеваемости врожденным гипотиреозом (на 10 000 новорожденных). 8.Показатели заболеваемости и распространенности ЙДЗ (структура заболеваемости щитовидной железы). 9.Показатель осложнений, вызванных ЙДЗ. 10.Показатель количества хирургических вмешательств по поводу ЙДЗ. 11.Охват индивидуальной йодной профилактикой групп риска развития ЙДЗ (беременные, кормящие, дети до 2 лет), %. 12.Число специалистов, подготовленных для работы по профилактике, диагностике и лечению ЙДЗ. 13.Количество новых технологий (патентов, методик) диагностики и лечения ЙДЗ. 14.Количество научно-практических мероприятий (конференций, семинаров, лекций) по вопросам профилактики и лечения ЙДЗ, проведенных для медицинских работников. 15.Количество подготовленных для населения информационных материалов по проблеме дефицита йода и ЙДЗ для печати (публикации, брошюры, плакаты и пр.) и размещения в сети Интернет; активность работы со СМИ (выступления/передачи на радио и телевидении), различными общественными организациями и обществами. 16.Показатель ожидаемой продолжительности жизни населения, страдающего ЙДЗ. 17.Показатель инвалидизации от ЙДЗ. 18.Показатель продолжительности сроков временной нетрудоспособности лиц с ЙДЗ. Сроки реализации Программы 202Х–202Х гг. Этапы основных мероприятий Программы I этап — 202Х год: 1.подготовка и проведение скринингового обследования населения с целью определения статуса йодной обеспеченности и оценки эпидемиологической ситуации по ЙДЗ в субъекте Российской Федерации (наименование); 2.проведение мероприятий, направленных на осуществление массовой йодной профилактики в субъекте; 3.проведение мероприятий групповой профилактики в контингентах повышенного риска (дети, подростки, беременные и кормящие женщины) II этап — 202Х–202Х гг. Реализация долгосрочной программы мероприятий по массовой и групповой профилактике, лечению ЙДЗ в субъекте Российской Федерации (наименование) III этап — 202Х–202Х годы Мониторинг результатов реализации Программы — проведение мероприятий по оценке статуса йодной обеспеченности населения и динамики эпидемиологической ситуации по ЙДЗ в субъекте Российской Федерации (наименование) Перечень основных мероприятий Программы 1.Создание централизованной службы профилактики, диагностики и лечения ЙДЗ в субъекте в виде организации региональных центров (кабинетов), действующих на функциональной основе на базе лечебно-диагностических подразделений «головного» ЛПУ по профилю «эндокринология» субъекта РФ или регионального эндокринологического центра (диспансера). 2.Оснащение центров (кабинетов) профилактики и лечения ЙДЗ современным оборудованием, расходными материалами и лекарственными средствами. 3.Проведение на постоянной основе лечебно-профилактических мероприятий, направленных на устранение ЙДЗ в субъекте РФ, и ведение мониторинга их эффективности в соответствии с разработанными стандартами. 4.Создание и обеспечение ведения регионального реестра ЙДЗ. 5.Организация и проведение среди населения субъекта (наименование) широкой разъяснительной работы по вопросам профилактики заболеваний, связанных с дефицитом йода, с использованием средств массовой информации. 6.Организация подготовки и повышения квалификации медицинского персонала центров по профилактике и лечению ЙДЗ. 7.Проведение научно-исследовательских работ, направленных на создание и внедрение новых методов профилактики, диагностики и лечения ЙДЗ. 8.Обеспечение насыщения рынка продовольственных товаров пищевой йодированной солью, использование ее в пищевой промышленности. 9.Информационное обеспечение населения, медицинских работников, врачей-специалистов соответствующими просветительскими и образовательными материалами по вопросам профилактики, диагностики и лечения ЙДЗ. Исполнители Программы и основных мероприятий 1.Орган исполнительной власти субъекта Российской Федерации в сфере здравоохранения (наименование). 2.Орган исполнительной власти субъекта Российской Федерации в сфере образования (наименование). 3.Территориальный орган санитарно-эпидемиологического надзора (наименование). 4.Территориальный фонд обязательного медицинского страхования (наименование). 5.Орган исполнительной власти субъекта Российской Федерации в сфере сельского хозяйства и продовольствия (наименование). 6.Орган исполнительной власти субъекта Российской Федерации в сфере связи и массовых коммуникаций (наименование). Объем и источники финансирования Общее финансирование на реализацию Программы 202Х–202Х гг.: тыс. руб. Источники. 1.Бюджет регионального уровня (бюджет субъекта Российской Федерации (наименование)). 2.Средства ОМС. 3.Средства внебюджетных фондов. Распределение финансирования Программы по годам 202Х г.: тыс. руб. 202Х г.: тыс. руб. 202Х г.: тыс. руб. 202Х г.: тыс. руб. 202Х г.: тыс. руб. Финансовые средства ежегодно уточняются в пределах имеющихся финансовых ресурсов. Ожидаемые конечные результаты реализации Программы Принятие и реализация региональных целевых программ на территории субъекта РФ позволят обеспечить население высококвалифицированной медицинской помощью, направленной на профилактику, раннюю диагностику и лечение ЙДЗ. В результате этого станет возможным: полностью ликвидировать синдром врожденной йодной недостаточности (эндемический кретинизм); увеличить продолжительность жизни населения, страдающего ЙДЗ, на 10–15 лет; сократить средний срок стационарного лечения пациентов с патологией щитовидной железы в 2–3 раза; cнизить инвалидизацию, связанную с ЙДЗ, на 30–40%; снизить количество хирургических вмешательств по поводу йододефицитных тиреопатий на 30%; уменьшить заболеваемость йододефицитными тиреопатиями (заболевания щитовидной железы) на 80%; снизить количество женского бесплодия, связанного с дефицитом йода, на 50–80%; уменьшить число осложнений у женщин, вызванных ЙДЗ во время беременности и в послеродовом периоде, на 20%, а также в раннем постнатальном периоде у новорожденных на 50%; уменьшить внутриутробную смертность за счет ранней диагностики гестационного гипотиреоза на 25%; своевременное выявление врожденного гипотиреоза позволит снизить инвалидизацию у детей на 100%. Контроль исполнения программы Система организации контроля за исполнением Программы включает исполнителей основных мероприятий в порядке, установленном для контроля за реализацией региональных целевых программ.

1.1. Основные понятия, используемые в настоящей целевой программе.

Для целей настоящей целевой программы используются следующие основные понятия:

1.2. Обоснование необходимости решения проблемы йододефицитных заболеваний.

Региональная целевая программа «Профилактика йододефицитных заболеваний на 202Х–202Х годы» разработана в рамках исполнения Постановления Правительства РФ от 26 декабря 2017 г. №1640 «Об утверждении государственной программы Российской Федерации “Развитие здравоохранения”», Постановления Правительства Российской Федерации от 05.10.1999. №1119 «О мерах по профилактике заболеваний, связанных с дефицитом йода» (с изменениями и дополнениями), Приказа Минздрава России от 15.01.2020 №8 «Об утверждении Стратегии формирования здорового образа жизни населения, профилактики и контроля неинфекционных заболеваний на период до 2025 года», Постановления Главного государственного санитарного врача РФ от 23.07.2008 №45 (ред. от 25.03.2019, с изм. и доп., вступ. в силу с 01.01.2020) «Об утверждении СанПиН 2.4.5.2409-08» («Санитарно-эпидемиологические требования к организации питания обучающихся в общеобразовательных учреждениях, учреждениях начального и среднего профессионального образования. Санитарно-эпидемиологические правила и нормативы»).

К ЙДЗ относят все патологические состояния, развивающиеся в популяции в результате дефицита йода (ЙД) в питании, которые могут быть предотвращены при нормальном потреблении йода.

ЙДЗ относятся к числу наиболее распространенных неинфекционных заболеваний во всем мире, представляя собой глобальную угрозу здоровью людей, требующую значительных ресурсов системы здравоохранения на их лечение.

Самым распространенным проявлением ЙДЗ является эндемический (диффузный нетоксический) зоб, являющийся предрасполагающим фактором для развития различных узловых форм заболеваний щитовидной железы (ЩЖ). Существует четкая зависимость между низким содержанием йода в пище и воде и развитием зоба у населения, а при поступлении йода в достаточном количестве наблюдается значительное уменьшение распространенности зоба.

Спектр йододефицитной патологии обширен и не ограничивается только заболеваниями ЩЖ. Дефицит йода оказывает неблагоприятное воздействие на рост и развитие организма еще во внутриутробном периоде, что проявляется нарушением формирования и развития ЦНС плода, прежде всего — головного мозга, с ограничением когнитивных и функциональных параметров развития у детей вплоть до выраженных форм умственной отсталости, повышенной частотой врожденных пороков развития и невынашивания беременности. Согласно заключению ВОЗ, недостаточность йода является самой распространенной причиной умственной отсталости, которую можно предупредить.

С начала 1970-х годов мероприятиям по профилактике ЙДЗ не уделялось достаточного внимания, что обусловило увеличение степени тяжести йодного дефицита и значительный рост заболеваемости. В 1990–2006 гг. сотрудниками ЭНЦ РАМН (в настоящее время ФГБУ «НМИЦ эндокринологии» Минздрава России) были проведены широкомасштабные эпидемиологические исследования, результаты которых показали, что в Российской Федерации практически нет регионов, где население не подвергалось бы риску развития ЙДЗ, а реальное потребление йода в 2–3 раза ниже рекомендуемого уровня. Данные ранее проведенных исследований подтверждаются результатами эпидемиологических исследований ФГБУ «НМИЦ эндокринологии» Минздрава России, выборочно выполняемых в регионах РФ, и свидетельствуют о недостаточном уровне йодной обеспеченности населения в условиях отсутствия массовой йодной профилактики: распространенность эндемического зоба у детей составляет в среднем 15–25%, а в некоторых регионах — до 40%.

Таким образом, эндемический зоб и другие ЙДЗ представляют собой актуальную медико-социальную проблему, которая может быть решена путем проведения адекватной йодной профилактики: популяционной (массовой) и индивидуальной.

За последние два десятилетия в мире был достигнут значительный прогресс в устранении нарушений, связанных с дефицитом йода. С 1990 по 2017 гг. число стран, имеющих дефицит йода в питании населения, сократилось с 113 до 20. Этот прогресс был обусловлен прежде всего расширенным внедрением программ йодирования соли.

Наиболее эффективным и безопасным методом решения проблемы дефицита йода в популяции (массовая профилактика) является йодирование пищевой поваренной соли, производимое путем добавления к обычной соли йодата калия (стандарт в России с 1998 г.). Йодирование соли является самой экономичной и устойчивой стратегией обеспечения оптимального потребления йода с питанием у всех групп населения.

Групповая йодная профилактика осуществляется в определенных группах населения повышенного риска развития ЙДЗ (беременные, кормящие матери, дети до 2 лет) с использованием препаратов йодида калия.

Необходимым мероприятием по продвижению знаний о проблеме йодного дефицита и методах его профилактики является широкомасштабная санитарно-просветительная и разъяснительная работа среди населения с использованием средств массовой информации.

1.3. Основные цели и задачи Программы.

Основная цель Программы — разработка и обеспечение функционирования системы профилактики, диагностики и лечения заболеваний, обусловленных дефицитом йода на территории субъекта Российской Федерации.

Основные задачи Программы.

1.Создание регионального центра (кабинета) профилактики и лечения ЙДЗ как одного из структурных подразделений системы специализированной централизованной службы профилактики, диагностики и лечения ЙДЗ, действующего на функциональной основе на базе лечебно-диагностического подразделения «головного» ЛПУ по профилю «эндокринология» субъекта РФ или регионального эндокринологического центра (диспансера).

2.Проведение системных контрольно-эпидемиологических исследований с целью оценки уровня дефицита йода в питании населения и распространенности ЙДЗ в субъекте РФ (наименование).

3.Проведение на постоянной основе лечебно-профилактических мероприятий, направленных на устранение ЙДЗ в субъекте РФ, и ведение мониторинга их эффективности в соответствии с разработанными стандартами.

4.Создание и регулярное ведение реестра по ЙДЗ в субъекте РФ.

5.Оснащение центров (кабинетов) профилактики и лечения ЙДЗ современным оборудованием, расходными материалами и лекарственными средствами.

6.Организация подготовки и повышения квалификации медицинского персонала центров (кабинетов) по профилактике и лечению ЙДЗ.

7.Проведение научно-исследовательских работ, направленных на создание и внедрение новых методов профилактики, диагностики и лечения ЙДЗ.

8.Информационное обеспечение населения, медицинских работников соответствующими просветительскими и образовательными материалами по вопросам профилактики, диагностики и лечения заболеваний, обусловленных дефицитом йода.

9.Способствование насыщению рынка субъекта Российской Федерации (наименование) йодированной солью и использованию ее в пищевой промышленности.

2.1. Определение эпидемиологии ЙДЗ.

Для определения распространенности ЙДЗ в субъекте РФ (наименование) необходимо проведение эпидемиологических исследований кластерным методом среди групп риска:

2.2. Создание/совершенствование противозобных центров (кабинетов).

Создание регионального центра (кабинета) профилактики и лечения ЙДЗ (как одного из структурных подразделений системы специализированной эндокринологической службы), профилактики, диагностики и лечения ЙДЗ необходимо для диспансерного наблюдения и лечения лиц с заболеваниями щитовидной железы, мониторинга профилактики ЙДЗ и результатов реализации Программы на уровне субъекта РФ.

2.3. Организация системы профилактики ЙДЗ и их осложнений.

Предусматривает создание оптимальной модели профилактики ЙДЗ с использованием йодированной соли, лекарственных средств с вовлечением различных региональных ресурсов всех уровней, включая:

2.4. Обеспечение эффективного лечения лиц с ЙДЗ.

Реализация этого направления подразумевает гарантированное обеспечение и прогнозирование потребности лиц, больных ЙДЗ, препаратами йодида калия и гормонов щитовидной железы с целью лечения в соответствии со стандартами оказания медицинской помощи и современными клиническими рекомендациями.

2.5. Обеспечение производства продуктов питания, содержащих йодированную соль.

Предусматривает принятие административных решений по организации производства пищевой продукции с использованием в рецептуре йодированной соли и реализации йодированной соли предприятиями продовольственной торговли на территории субъекта РФ (наименование).

Программой предусмотрен план мероприятий по ее реализации (приложение №1).

3.1. Гарантированное обеспечение населения субъекта РФ (наименование) препаратами для профилактики и лечения ЙДЗ.

Для гарантированного обеспечения населения групп риска и больных ЙДЗ препаратами для профилактики и лечения ЙДЗ, прогнозирования объемов закупок и оптимизации затрат бюджетов всех уровней необходимо:

3.2. Организация профилактики и лечения ЙДЗ субъекта РФ.

С целью создания, развития и совершенствования противозобной службы субъекта РФ необходимо организовать на базе ЛПУ (наименование) региональный центр профилактики и лечения ЙДЗ (противозобный центр или кабинет).

Задачи регионального центра профилактики и лечения ЙДЗ субъекта РФ:

Для выполнения указанных задач необходимо:

3.3. Освещение проблемы йододефицитных заболеваний в средствах массовой информации.

Для повышения информированности населения о проблеме дефицита йода в питании и мерах по профилактике ЙДЗ необходимо проводить санитарно-просветительную и широкомасштабную информационно-разъяснительную работу среди населения субъекта РФ (наименование) через средства массовой информации (телевидение, радио, пресса, сеть Интернет), уделяя особое внимание методам йодной профилактики с регулярным использованием в питании йодированной соли.

3.4. Совершенствование системы гигиенического мониторинга.

Для осуществления контроля за реализацией Программы в субъекте РФ (наименование), ее эффективностью необходимо создание постоянно действующего гигиенического мониторинга.

Мониторинг эффективности профилактических программ осуществляется путем организации непрерывной оценки обеспеченности населения йодом. Критериями эффективности программ массовой йодной профилактики (на основании рекомендаций ВОЗ и ЮНИСЕФ) являются:

Основным эпидемиологическим показателем, характеризующим обеспеченность йодом жителей региона, является медианная концентрация йода в моче (медиана йодурии, МКЙ). Медиана йодурии — высокочувствительный показатель, быстро реагирующий на изменения в уровне потребления йода, имеет важнейшее значение не только для оценки эпидемиологической ситуации, но и для осуществления контроля программ профилактики ЙДЗ. Выбор репрезентативной группы (включает детей допубертатного возраста 8–10 лет) для оценки йодной обеспеченности в популяции осуществляется кластерным методом, что является наиболее эффективным и обоснованным с практической точки зрения.

Целесообразно изучить охват йодированной солью на уровне домохозяйств или обеспеченность питания населения йодом на уровне отдельных географических территорий (районов) и социально-экономических групп.

Результаты неонатального скрининга на гипотиреоз используются в оценке распространенности и степени тяжести ЙДЗ. На основании этих результатов можно предположить наличие в данной среде факторов, влияющих на щитовидную железу, а именно обеспеченность йодом. У новорожденных ТТГ находится в обратной зависимости с уровнем йодурии. Дефицит йода у матери — самая частая причина повышения уровня ТТГ у новорожденных в йододефицитных районах. Всемирная организация здравоохранения (ВОЗ) в 1994 г. определила критерии неонатальной гипертиреотропинемии для регионов с легким, умеренным и тяжелым йодным дефицитом. Согласно рекомендациям, для территорий с благополучным йодным обеспечением уровень неонатального ТТГ выше 5 мкМЕ/л определяется не более чем у 3% новорожденных, в регионах с легким йодным дефицитом этот показатель составляет 3–19,9%, с умеренным — 20–39,9%. Показатель неонатального ТТГ выше 5 мкМЕ/л для оценки йододефицита имеет и финансовые преимущества, ведь он охватывает всех новорожденных на данной территории и не требует дополнительных исследований.

Дополнительным показателем является распространенность зоба у детей — эпидемиологический показатель состояния йодного дефицита, который в большей степени отражает результаты долгосрочных профилактических мероприятий (а не текущую ситуацию).

Программа реализуется за счет средств бюджета субъекта РФ (наименование) и средств территориального фонда обязательного медицинского страхования.

Общий объем финансирования мероприятий и его распределение по годам реализации программы, включая скрининговые исследования, улучшение диагностической базы, обеспечение лекарственными препаратами, указаны в приложении №Х.

Финансовые средства на реализацию Программы ежегодно уточняются в установленном порядке.

Выделенные бюджетные средства для реализации мероприятий Программы распределяются заказчиком на конкурсной основе посредством заключения государственных контрактов (договоров).

Заказчик при необходимости может обращаться в администрацию субъекта РФ (наименование) и территориальный фонд обязательного медицинского страхования с запросом о выделении дополнительных средств.

Контроль за выполнением Программы осуществляется заказчиком (Орган исполнительной власти субъекта Российской Федерации в сфере здравоохранения (наименование)) совместно с территориальным органом санитарно-эпидемиологического надзора субъекта Российской Федерации (наименование).

Эффективность реализации Программы оценивается исходя из показателей эффективности проведения профилактических мероприятий и лечения больных ЙДЗ:

## ДОПОЛНИТЕЛЬНАЯ ИНФОРМАЦИЯ

Источники финансирования. Работа выполнена в рамках государственного задания Минздрава России «Эпидемиологические и молекулярно-клеточные характеристики опухолевых, аутоиммунных и йододефицитных тиреопатий как основа профилактики осложнений и персонализации лечения», рег. № АААА-А20-120011790180-4.

Конфликт интересов. Авторы декларируют отсутствие явных и потенциальных конфликтов интересов, связанных с публикацией настоящей статьи.

Участие авторов. Трошина Е.А., Платонова Н.М., Беловалова И.М., Мельниченко Г.А., Дедов И.И. — концепция и дизайн исследования; Маколина Н.П., Сенюшкина Е.С. — обработка материалов, анализ полученных данных, написание текста; Дедов И.И. — системное научно-методологическое и практическое руководство на всех этапах создания проекта программы. Все авторы одобрили финальную версию статьи перед публикацией, выразили согласие нести ответственность за все аспекты работы, подразумевающую надлежащее изучение и решение вопросов, связанных с точностью или добросовестностью любой части работы.

